# Micro-perspective of listed companies in China: Digital development promotes the green transformation of the manufacturing industry

**DOI:** 10.1371/journal.pone.0293474

**Published:** 2023-10-26

**Authors:** Haohui Wang, Lunwen Wu, Gang Peng, Hongmei Du

**Affiliations:** 1 School of Statistics, Southwestern University of Finance and Economic, Chengdu, Sichuan Province, China; 2 School of Business Administration, Southwestern University of Finance and Economics, Chengdu, Sichuan Province, China; 3 School of Public Administration, China University of Geosciences, Wuhan, Wuhan City, Hubei Province, China; East China Normal University, CHINA

## Abstract

In the context of the rapid development of the global digital economy, it is of great significance to explore the greening transformation of the manufacturing industry from the micro-perspective of enterprise digital development. This paper empirically examines the impact and mechanism of enterprise digital development on the greening transformation of the manufacturing industry using the 2010–2020 data of Chinese A-share listed companies in the manufacturing industry as a sample. The study shows that enterprise digital development can significantly promote the greening transformation of China’s manufacturing industry, and this conclusion still holds after a series of robustness tests. Technological innovation and financing constraints are important mediating mechanisms. Further research found that the impact of enterprise digital development on the greening transformation of China’s manufacturing industry has a positive nonlinear effect, and its marginal effect shows a weakening trend. Heterogeneity analysis shows that, from the perspective of micro characteristics, digital development is more able to promote the green transformation of state-owned and large enterprises. From a macro-regional perspective, digital development can better promote the green transformation of the manufacturing industry in eastern cities, key city clusters, and high-level cities. The findings of this paper can provide corresponding insights for "revitalizing the manufacturing industry", and also provide decision-making references for countries aiming to make the manufacturing industry bigger and stronger.

## Introduction

Limited by increasingly severe environmental problems and resource constraints, green transformation and sustainable development have become global consensus issues [1]. Take China as an example. At the 75th General Debate of the United Nations General Assembly, President Xi Jinping proposed that China should strive to peak its carbon dioxide emissions by 2030 and work towards carbon neutrality by 2060.

Manufacturing is an important pillar of the world’s economic development. Under the new situation, "revitalizing the manufacturing industry" has become an important strategic policy for leading economies. Taking China as an example, "from big to strong" is the primary goal of its manufacturing development. As a major energy consumer in economic operations, the development and greening of the manufacturing sector is an important part of the global economy.

With the booming development of the digital economy (De), the new generation of digital technologies is driving the manufacturing industry to make profound changes in all aspects of the value creation process. As of 2021, the scale of the value added of the global De is $38.1 trillion, accounting for 45.0% of GDP. From the perspective of the world pattern, the scale of De of the dominant economies reached 27.6 trillion U.S. dollars, accounting for 72.5% of the global total. The United States, China, and the European Union form a three-polar pattern of global De, in which the United States leads the world in the scale of De, amounting to 15.3 trillion U.S. dollars, and China’s 7.1 trillion U.S. dollars rank second in the world. Against this backdrop, economies around the world have placed digitization and greening in a highly strategic position. The United States has released the National Network Strategy, the United Kingdom has introduced the Digital Development Strategy, and Germany has implemented the "Industry 4.0" development strategy. In China, for example, the concept of "greening" was first proposed at the meeting of the Political Bureau of the Central Committee in 2015, and a "dual-carbon" target was proposed for 2020.

To summarize, digital development and green transformation are causing profound changes, whether in top-level national strategies, regional development at the provincial and city levels, or in the ecological landscape of the industry.

In 2020, China’s State-owned Assets Supervision and Administration Commission (SASAC) issued the Notice on Accelerating the Digital Transformation of State-owned Enterprises, which comprehensively deploys the digital transformation of state-owned enterprises (Dig). Based on this, a question of theoretical and practical value has become more and more prominent: from the micro level, how does the digital development of enterprises affect the greening transformation of the manufacturing industry (Gtf)? Unfortunately, there is little literature on this important proposition for in-depth study.

## Literature review

In this paper, we review the research literature related to Dig as well as Gtf.

### Literature related to enterprise digitization

Early research on Dig mainly focused on discussing its definition, connotation, and other aspects. European and American scholars have defined Dig with more emphasis on the computer and technological characteristics of digitization, focusing on the advantages and development of technology [2, 3].Chinese scholars tend to define Dig in terms of "emerging digital technology + physical enterprise", focusing on the practical application of the technology [4, 5]. According to recent study, Dig is defined as the deep integration of mobile Internet, Internet of Things, big data, cloud computing, artificial intelligence, and physical enterprises [6].

With the concept of Dig becoming clearer and more developed, research related to Dig is gradually turning to the economic benefits it brings. Studies have focused on the competitive strategy, enterprise innovation, total factor productivity, labor share, corporate governance, and other aspects of business development [7–9]. For example, some literature suggests that Dig has a positive impact on human capital, operational capability, and investment efficiency [10]. Some literature suggests that Dig can help enterprises improve their innovation level and labor productivity and ultimately obtain more economic benefits [11].

In the recent research literature related to Dig, scholars have extensively explored the contribution of Dig to environmental protection. Part of the literature starts from the perspective of energy savings and emission reduction and establishes the DID model to explore the contribution of Dig to enterprise pollution reduction [12]. Part of the literature focuses on the environmental, social, and corporate governance (ESG) performance. For example, some scholars have subdivided Dig into five components: digital resources, organization, adoption, innovation culture, and digital management, and explored the facilitating effect of each path on enterprise ESG, respectively [13]. Part of the literature, on the other hand, directly proposes that the intelligence of enterprises can effectively promote Gtf [14].

According to the literature combing, Dig has gradually tended to be standardized, and the related research has shifted from the initial definition research, economic benefit research, to environmental benefit research.

### Literature related to greening transformation of manufacturing industry

The academic community has extensively explored various aspects of factors affecting Gtf.

In the early research literature, scholars focused more on exploring the impact of policy elements or infrastructure development on Gtf. Meanwhile, among the studies on policy elements, green credit policy and environmental regulation are the focus of research. For example, Lee proposed that a green credit policy can significantly reduce the carbon emission intensity of the manufacturing industry through capital renewal [15]. Part of the literature combines environmental regulations (ER) with anti-corruption policies (AC) and analyzes that ER implementation is more effective when AC is at a higher level in the process of greening manufacturing [16]. Part of the literature categorizes government policies into five types: fiscal, tax, financial, technological, and environmental, and suggests that coordinated policies have a higher level of effectiveness on the green innovation performance of the manufacturing industry than using separate policies [17]. Part of the literature approaches from the perspective of infrastructure development, arguing that infrastructure development can facilitate Gtf through scale effects and energy restructuring [18].

With the development of Dig, scholars have begun to focus on the impact of digital technologies on Gtf. Digital technologies specifically include big data, smart manufacturing, robotics, and artificial intelligence, all of which are closely related to manufacturing. Some studies have focused on the deep integration of manufacturing and big data, proposing that big data can improve the factor allocation rate of labor and capital and ultimately promote the green development transformation of manufacturing [19]. Part of the literature examines the facilitating role of intelligent manufacturing in green innovation, proposing that manufacturing intelligence is conducive to the "technology facilitation effect" and the "cost reduction effect", thus promoting green technological innovation [20, 21]. Part of the literature reveals the facilitating effect of industrial robotics (IRA) application on green technology innovation (GTI) in global manufacturing [22].

To summarize, at the initial stage of Gtf, factors such as policy and infrastructure were key to Gtf. With the deepening of policy practices and the improvement of infrastructure, the importance of the technological dimension has gradually emerged, Dig has become another key factor in Gtf.

### Literature summary and contributions

Existing studies may have the following shortcomings: First, most of the current literature measures and analyzes the level of De at the macro level, and although there is a lot of literature on the concept of Dig that qualitatively analyzes and combs the concept of Dig, there is relatively little literature that measures Dig from a quantitative perspective. Second, there is less literature discussing the greening transition from a micro perspective, especially from the perspective of enterprise carbon emission reduction efficiency. Third, the existing literature on the relationship between digitization and greening transformation is relatively small, and in the few relevant literatures, most of them discuss greening transformation based on the more macro and broad perspectives, such as big data and smart manufacturing, and there is a serious lack of research arguments on Dig at the micro level on Gtf.

The possible contributions are in the following areas:

First, perspective addition. Based on the micro data of listed companies in China, a discussion on the impact of Dig on corporate carbon efficiency provides a nuanced micro perspective on Gtf.

Second, innovation in indicator construction. In terms of Dig, the current literature on measuring Dig at the microlevel is relatively small. This paper constructs a unique digitization thesaurus based on policy documents from the official websites of China’s Central People’s Government (CPG) and China’s Ministry of Industry and Information Technology (MIIT), using Python text analysis technology. On the one hand, starting from the annual reports of listed companies, we measure the word frequency related to enterprise digitization based on the thesaurus. On the other hand, starting from the financial data of enterprises, we extract the digitized intangible assets of enterprises based on the keywords of the thesaurus. The degree of Dig is measured in different dimensions.

On the measurement of Gtf, this paper projects corporate carbon emissions through manufacturing industry data. On the one hand, from the single-factor idea, the carbon emission efficiency of enterprises is measured according to the ratio of enterprise business income and enterprise carbon emissions, so as to define Gtf. On the other hand, from a full-factor perspective, the SBM-GML model and the EBM-GML model are applied to measure Gtf.

On the measurement of environmental regulation, this paper constructs a thesaurus of environmental regulation based on the government work report of the State Council using Python text analysis technology and does word frequency analysis of the corresponding keywords in the government work report of each prefecture-level city according to the thesaurus so as to quantify environmental regulation.

Third, this paper provides more diverse and robust arguments and conclusions for Gtf. First, this paper explores the direct impact of Dig on Gtf and conducts a series of robustness tests by introducing lagged terms, instrumental variables, quasi-natural experiments, and replacing various indicators. Secondly, this paper combs through the theoretical mechanisms and influence of Dig on Gtf from the perspectives of technological innovation and financing constraints. Further, this paper provides a nonlinear discussion on the spillover effect of digitalization. Finally, the heterogeneity analysis is carried out from the perspectives of microenterprise characteristics and macroregional characteristics, respectively, in an attempt to analyze the impact of Dig on Gtf in a more multidimensional and comprehensive way.

## Theoretical mechanisms and hypotheses

Dig is a transformation process for enterprises centered on digital technology that creates greater economic benefits while inherently enhancing environmental sustainability [23]. Dig mainly promotes Gtf from three aspects: production factors, production process, and end-end monitoring.

First, at the production factor end, Dig can improve resource allocation efficiency and production material use efficiency [24]. Second, in the production process, digitalization can promote the harmless production of enterprises. Enterprises using digital technology can change the traditional manufacturing industry’s high-pollution, high-energy-consuming production path, which in turn reduces pollution emissions and realizes green transformation [25]. Finally, in terms of end-of-pipe monitoring, first, digital technology can more accurately and efficiently identify the types and characteristics of discharged wastes, improve the efficiency of waste disposal, and realize a sustainable supply chain [26, 27]. Second, digital infrastructure can more accurately predict the pollution risk of enterprises, improve the control system of pollution, and reduce the impact of unexpected pollution events [28]. Based on this, this paper proposes:

H1: Dig can effectively promote Gtf.

On the one hand, Dig can prompt the manufacturing industry to carry out green technological innovation. Through the introduction of digital transformation methods and digital technologies, the management ability of enterprises to acquire, utilize, and coordinate internal and external resources is strengthened [29]. During the production process, the efficient digital management capability of the enterprise effectively reduces the cost of conducting green innovation research and development, which increases the enterprise’s green innovation initiative and makes it easier to improve the quantity and quality of green innovation [30]. Meanwhile, the higher the degree of Dig, the more they tend to increase R&D expenditures, which in turn promotes green innovation output [31].

On the other hand, green innovation technology can accelerate Gtf. The use of non-renewable energy continues to dominate the development of the manufacturing sector at this time. Change the traditional technological innovation model; the use of green innovation technology that can achieve sustainable development and harmony between human beings and nature is an important way to break this status quo [32]. The innovation effect of green technology can improve resource efficiency, and reduce production costs. It is conducive for enterprises to identify waste in the production and operation processes, adjust the energy structure, promote energy savings and emission reduction, and realize green development [33]. At the same time, green technology is characterized by strong systematicity, a focus on ecological prevention, economic compliance, and improved efficiency. Utilizing green technology helps to increase the social capital needed for the future development of enterprises and ensure corporate image, business goals, and competitive advantages. The motivation for Gtf will be enhanced as a result [34]. Based on this, this paper proposes:

H2: Dig promotes Gtf by improving technological innovation capabilities.

Gtf is closely related to the financing constraints of enterprises, and many enterprises face the dilemma of hindered transformation due to their inability to obtain sufficient external funds [35]. Under the traditional model, investors will take a more cautious attitude towards enterprise credit businesses based on the principles of profitability and security, thus exacerbating the financing constraints of enterprises [36].

Dig helps to promote enterprise information transparency, which is one of the effective solutions for enterprises to alleviate the problem of financing constraints. According to signaling theory, Dig is a positive signal sent outward by enterprises, indicating that they have good development strength and potential when they are more competitive in the financing market. At the same time, enterprise managers can not only use digital technology to obtain enterprise information efficiently but also transmit information outward at low cost on the digital platform, effectively alleviating the problem of internal and external information asymmetry [37]. According to the long-tail effect, a large number of small customer groups are unable to obtain adequate financing in financial markets, and Dig can improve communication between tail manufacturing enterprises and investors. With the help of digital platforms, enterprises can dock with financial institutions and many social capitals, and new investors and financing channels effectively alleviate the financing constraints of tail enterprises and improve the speed and efficiency of financing [38]. The alleviation of financing constraints can better promote enterprise R&D investment and technological innovation, thus effectively promoting Gtf. Based on this, this paper proposes:

H3: Dig promotes the Gtf by alleviating financing constraints.

Dig has changed their production logic to a certain extent, which may have non-linear spillover effects on their productivity and Gtf. On the one hand, Dig improves the efficiency of resource utilization, which helps enterprises carry out large-scale production and realize the scale effect. On the other hand, Dig can also achieve clean production, energy savings, emission reduction, and environmental pollution reduction [39]. At the same time, Dig has cyclical characteristics. Only through gradual accumulation can digital technology be integrated into existing research and development, production, operation, management, and other aspects and ultimately realize the technological change of industrial digitalization [40]. Therefore, the impact of Dig is also characterized by phases. In the early stage of Dig, the technology integration itself is not perfect; the scale effect of the enterprise dominates, and the effect of pollution increase is greater than the reduction effect at this time. However, with the continuous improvement of technology, the pollution reduction effect continues to improve, coupled with the entrepreneur’s pursuit of social responsibility, the enterprise’s environmental performance continues to improve, and ultimately the green transformation [39]. Based on this, this paper proposes:

H4: Dig has a nonlinear spillover effect on Gtf.

Cities vary in terms of regional location, economic development, natural resource conditions, etc., and there are also differences in size and attributes among businesses. Based on this, the degree of Dig and the possible impact on the environment also differ. In terms of initial endowment, natural resource conditions vary greatly between eastern and western China: western China is a major producer of raw coal and crude oil, whereas eastern China lacks natural resources and produces almost no raw coal or crude oil. Meanwhile, the imbalance of economic in each region also makes the investment in human resources and technology vary greatly among different enterprises [41]. On this basis, Dig may exacerbate these disparities. In more economically developed regions, the construction of digital infrastructure is more complete, and digital technology can effectively break through the limitations of time and space, promote the efficient flow of production factors, reduce production costs, and help enterprises achieve pollution reduction and green transformation. In economically backward regions, capital, factors, talents, and other factors cannot be effectively configured. Digital technology is conducive to enterprises achieving the scale effect and the pursuit of greater economic benefits, but it is difficult to promote Gtf [42]. Based on this, this paper further proposes:

H5: There is a heterogeneous impact of Dig on Gtf.

**Fig 1:**
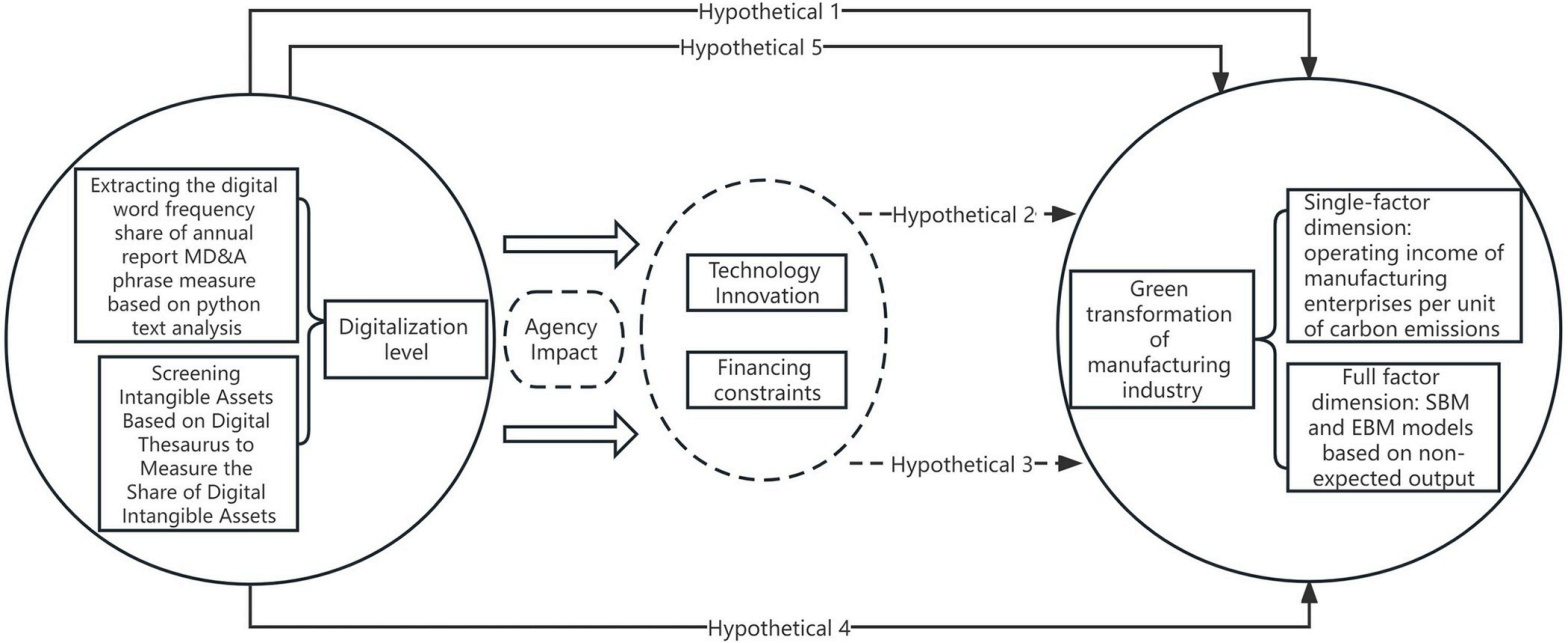
Flow chart of theoretical mechanism and research hypothesis.

The above theoretical mechanisms are summarized and the research hypotheses are formulated as shown in Fig 1.

## Materials and methods

### Model

#### Baseline model

This paper constructs the following Eq (1) to test the impact of Dig on Gtf [43]:

$$Gtf_{it}=\alpha +\beta Dig_{it}+\gamma Controls_{it}+\mu_{p}+\delta_{I}+\lambda_{Y}+\varepsilon_{it}$$ (1)

The explanatory variable Gtf_it_ represents the level of greening transformation of manufacturing enterprise i in year t. Digit is the core explanatory variable, representing the digitization level of enterprise i in year t. Controlsit is a series of control variables, including macro-level environmental regulation, urban development level, and micro-level indicators such as enterprise years, enterprise size, financial leverage, and rate of return. In order to control for the impact of macro and industry factors on Gtf, province fixed effects μ_p_, industry fixed effects δ_I_, and year fixed effects λ_Y_ are added to the equation. To make the statistical inference results more robust, this paper adopts robust standard errors to estimate the regression model.

#### DID and parallel trend tests

The State Council of China started to lay out the Broadband China strategy in 2013. Since then, a total of 120 cities (clusters) have been selected as "Broadband China" demonstration sites in 2014, 2015, and 2016. The demonstration areas will accelerate the improvement of information infrastructure construction and significantly increase local broadband coverage. The "Broadband China" policy (BDC) is used as a proxy variable for Dig for endogeneity testing [43], and the complete DID model is shown in Eq (2). The indicator construction rules are as follows: listed companies whose city of registration is a pilot city take the value of 0 in the year before the policy and take the value of 1 in the current year and the following years; the rest of the listed companies always take the value of 0.


$$Gtf_{it}=a_{0}+a_{1}BDC+\beta_{1}Controls_{it}+\mu_{p}+\delta_{I}+\lambda_{Y}+\varepsilon_{it}$$ (2)

The validity of the DID results depends on the parallel trend hypothesis test. The parallel trend test model is shown in Eq (3), and we use the event study method to test whether the model satisfies the parallel trend assumption. Where BDC_pre_s_ is a dummy variable indicating the s year before the pilot city enters the BDC. BDC_current_ indicates the year in which the pilot city enters the BDC. BDC_post_s_ indicates the s year after the pilot city enters the BDC. The longest duration of the policy in the pilot cities during the sample period is 6 years (corresponding to the first batch of BDC cities), and the longest duration of the policy in the pilot cities before implementation is 6 years (corresponding to the third batch of BDC cities). Prior periods longer than 4 years are combined as pre_4, and we set pre_4 as the base period. If the coefficients of the BDC_pre_s_ are statistically insignificant, the parallel trend hypothesis holds [44].


$$Gtf_{it}=b_{0}+\sum_{s=1}^{6}b_{pre\_s}BDC_{pre\_s}+b_{current}BDC_{current}+\sum_{s=1}^{6}b_{post\_s}BDC_{post\_s}+\beta_{2}Controls_{it}+\mu_{p}+\delta_{I}+\lambda_{Y}+\varepsilon_{it}$$ (3)

#### Channel and mechanism analysis

In the theoretical mechanisms and hypotheses section, we theoretically analyzed that Dig can promote Gtf through green technology innovation and financing constraints, but the issue has not been confirmed. Therefore, in order to further explore this issue, this paper empirically tests the influence mechanism by using the mediation effect model [45]. The complete mediation effect model is Eqs (4), (5) and (6):

$$Gtf_{it}=c_{0}+c_{1}Dig_{it}+\beta_{3}Controls_{it}+\mu_{P}+\delta_{I}+\lambda_{Y}+\varepsilon_{it}$$ (4)

$$Int_{it}=d_{0}+d_{1}Dig_{it}+\beta_{4}Controls_{it}+\mu_{P}+\delta_{I}+\lambda_{Y}+\varepsilon_{it}$$ (5)

$$Gtf_{it}=e_{0}+\phi Int_{it}+e_{1}Dig_{it}+\beta_{5}Controls_{it}+\mu_{P}+\delta_{I}+\lambda_{Y}+\varepsilon_{it}$$ (6)

Where Int denotes the mediating variable.

#### Nonlinear spillover effects

The technological characteristics of Dig have considerable combined coverage and penetration, a characteristic that makes it likely to have nonlinear threshold effects. In this paper, a panel threshold regression model is used to test the nonlinear spillover effects. Thresholds were estimated by repeated sampling 300 times using bootstrapping [46]. The panel threshold model is shown in Eq (7) [47, 48]:

$$Gtf_{it}=f_{0}+f_{1}Dig_{it}I(M_{it}\leq Z_{1})+f_{2}Dig_{it}I(Z_{1}<M_{it}\leq Z_{2})+\cdots +f_{n+1}Dig_{it}I(M_{it}>Z_{n})+\mu_{P}+\delta_{I}+\lambda_{Y}+\varepsilon_{it}$$ (7)

Where M_it_ is the threshold variable, Z is the threshold value and I(•) is the indicator function.

### Variables

#### Greening of manufacturing transformation

(1) This article measures Gtf with the carbon emission economic efficiency of manufacturing enterprises (hereinafter referred to as enterprise carbon efficiency). The carbon emissions of enterprises are estimated based on the carbon emissions of industries, and the ratio of business income to carbon emissions is used to measure the carbon efficiency of enterprises [49].
(2) In the robustness test, this paper adopts the Super–SBM model to measure Gtf. Suppose there are n decision-making units DUM, $DUM_{j}(j=1,2,\cdots ,n)$. Each DUM has m inputs, q1 desired outputs as well as q2 non-desired outputs, then the inputs, desired outputs as well as non-desired outputs are $x_{i}(i=1,2,\cdots ,m)$, $y_{k}^{g}(k=1,2,\cdots ,q_{1})$, $y_{l}^{b}(l=1,2,\cdots ,q_{2})$. The expression of the Super–SBM model is as follows (Eqs (8) and (9)) [50]:

$$\rho =min\frac{1+\frac{1}{m}\sum_{i=1}^{m}\frac{s_{i}^{-}}{x_{io}}}{1-\frac{1}{q_{1}+q_{2}}(\sum_{k=1}^{q_{1}}\frac{s_{k}^{g}}{y_{ko}^{g}}+\sum_{l=1}^{q_{2}}\frac{s_{l}^{b}}{y_{lo}^{b}})}$$ (8)

$$s.t.\{\begin{array}{c} x_{io}\geq \sum_{j=1,\neq o}^{n}\lambda_{j}x_{ij}-s_{i}^{-},\;\forall i;\\ y_{ko}^{g}\leq \sum_{j=1,\neq o}^{n}\lambda_{j}y_{kj}^{g}+s_{k}^{g},\;\forall k;\\ y_{lo}^{b}\geq \sum_{j=1,\neq o}^{n}\lambda_{j}y_{lj}^{b}-s_{l}^{b},\;\forall l;\\ 1-\frac{1}{q_{1}+q_{2}}(\sum_{k=1}^{q_{1}}\frac{s_{k}^{g}}{y_{ko}^{g}}+\sum_{l=1}^{q_{2}}\frac{s_{l}^{b}}{y_{lo}^{b}})>0;\\ s_{i}^{-}\geq 0,s_{k}^{g}\geq 0,s_{l}^{b}\geq 0,\lambda_{j}\geq 0,\forall i,k,l,j \end{array}$$ (9)

where *ρ* is the efficiency value of the Super–SBM model. $s_{i}^{-}$, $s_{k}^{g}$, and $s_{l}^{b}$ denote the slack variables for inputs, desired outputs, and non-desired outputs, respectively. *λ* is the weights of inputs and outputs. The subscript o denotes the DUM being estimated. Capital inputs in the model are measured by the firm’s total fixed assets; labor inputs are measured by the firm’s number of employees; desired outputs are defined as operating revenues; and undesired outputs are defined as the firm’s carbon emissions.

This study utilizes the Super–SBM model to assess the greening transformation of manufacturing industry, in combination with the GML index, in order to enhance the precision of measurement outcomes. Eq (10) depicts the GML index [51, 52]. The calculation of the GML index requires the construction of T+1 production possibility sets, T current production possibility sets, and 1 global production possibility set. Assuming a production efficiency of θ, the GML index is defined as follows:

$$\begin{array}{l} GML^{t,t+1}=\frac{\theta^{G,t+1}(x^{t+1},y^{t+1},b^{t+1})}{\theta^{G,t}(x^{t},y^{t},b^{t})}\\ =\frac{\theta^{t+1}(x^{t+1},y^{t+1},b^{t+1})}{\theta^{t}(x^{t},y^{t},b^{t})}*\frac{\theta^{G,t+1}(x^{t+1},y^{t+1},b^{t+1})/\theta^{t+1}(x^{t+1},y^{t+1},b^{t+1})}{\theta^{G,t}(x^{t},y^{t},b^{t})/\theta^{t}(x^{t},y^{t},b^{t})}\\ =\frac{TE^{t+1}}{TE^{t}}*\frac{BPG^{G,t+1}}{BPG^{G,t}}\\ =EC^{t,t+1}*TC^{t,t+1} \end{array}$$ (10)

where TE is the technical efficiency of the current set of production possibilities and BPG is the technical efficiency gap measured by the global set of production possibilities and the current set of production possibilities. At this point, the GML index can also be divided into two components: the technical progress index (TC) and the technical efficiency index (EC), both of which are greater than 1, reflecting technical progress and efficiency improvements, respectively. If the GML exceeds 1, it indicates an improvement in Gtf.

(3) In the robustness test, this paper also uses the EBM–GML model to measure Gtf as a proxy variable. The EBM–GML model is constructed as Eqs (11) and (12):

$$\gamma^{*}=\min\frac{\theta -\varepsilon_{x}^{}\sum_{i=1}^{m}\frac{\omega_{i}^{-}s_{i}^{-}}{x_{ik}}}{\phi +\varepsilon_{y}^{}\sum_{r=1}^{s}\frac{\omega_{r}^{+}s_{r}^{+}}{y_{rk}}+\varepsilon_{b}^{}\sum_{p=1}^{q}\frac{\omega_{p}^{b-}s_{p}^{b-}}{b_{pk}}}$$ (11)

$$s.t.\{\begin{array}{c} \sum_{i=1}^{m}x_{ij}\lambda_{j}+s_{i}^{-}=\theta x_{ik},\;i=1,.,m\\ \sum_{r=1}^{s}y_{ij}\lambda_{j}-s_{r}^{+}=\phi y_{rk},\;r=1,.,s\\ \sum_{p=1}^{q}b_{ij}\lambda_{j}+s_{p}^{b-}=\phi b_{pk},\;p=1,.,q\\ \lambda_{j}\geq 0,s_{i}^{-},s_{r}^{+},s_{p}^{b-}\geq 0 \end{array}$$ (12)

In the above equation, γ* is the optimal efficiency value of the EBM model. The inputs i, desired outputs r, and non-desired outputs p of the decision-making unit (DMU) k are denoted by *x*_*ik*_, *y*_*rk*_, and *b*_*pk*_, respectively. $s_{i}^{-}$, $s_{r}^{+}$, and $s_{p}^{b-}$ denote the slack variables of the input factor i, the desired outputs r, and the non-desired outputs p. $\omega_{i}^{-}$, $\omega_{r}^{+}$, and $\omega_{p}^{b-}$ denote the weights of the inputs i, the desired outputs r, and the non-desired outputs p, respectively. θ is the radial part of the planning parameter, and ϕ is the radial part of the planning of the output indicator parameter. *ε*_*x*_, *ε*_*y*_ and *ε*_*b*_ denote the core parameters of the radial component planning parameter θ and the non-radial slack variables $s_{i}^{-}$, $s_{r}^{+}$ and $s_{p}^{b-}$, respectively.

*λ* is a linear combination factor. When γ* = 1 is running, this DMU is technically efficient compared to other DMUs. The GML index is as described earlier.

### Enterprise digitization level

(1) Python text analysis method [37]. Dig in this paper are based on the collaboration of three steps: digitized vocabulary construction, text analysis of listed companies’ annual reports, and digitization index measurement.The first step is digitization and word set construction.

The first step is the construction of a digitized lexicon. As an emerging concept in recent years, there is no authoritative standard thesaurus for digitization in current practice. In order to accurately measure Dig, this paper attempts to construct a comprehensive and authoritative digitization lexicon applicable to China.

First, a policy search was conducted on the official websites of the Central People’s Government of China and the Ministry of Industry and Information Technology (MIIT) to screen out a total of 51 policy documents related to De as of August 2022. Second, the 51 policy documents are put into Python for text segmentation, and Python deactivation is applied to remove irrelevant words in the text. Through manual identification, 430 words related to De in the policy documents that do not exist in the original Python thesaurus are identified and added to the "jieba" thesaurus. Finally, the new thesaurus was used to perform word segmentation and word frequency statistics on 51 policy documents, and 336 words with a frequency of more than 5 times were selected to form a digitized lexicon.

In the second step, the textual analysis of corporate annual reports is conducted. Considering the changes in annual report disclosure format before and after 2014, we manually extracted the "Board of Directors’ Report" (before 2014) or "Management Discussion and Analysis" (after 2014) part of the annual reports and recorded the total number of words in the extracted part. A new thesaurus with 336 authoritative digitized words was used to analyze the extracted content of each enterprise’s annual report, and the frequency of occurrence of the 336 digitized words in each enterprise’s annual report was counted.

The "Report of the Board of Directors" or "Management’s Discussion and Analysis" section is usually the disclosure of information about the company’s future business development, risk management strategy, future development strategy, etc. It is more likely to be used to describe the company’s digitization-related content, and the analysis of this section can be used to construct Dig in a more reasonable and effective way.

The third step is the measurement of Dig. After obtaining the word frequencies of 336 words in the annual reports of enterprises, considering the differences in the length of the selected paragraphs of each annual report, this paper takes the sum of the word frequencies of the De words in the annual reports and divides it by the total length of the selected paragraphs of the annual reports to express Dig in that year. Considering the convenience of data expression, this paper multiplies the obtained ratio by 100 to represent the final Dig index.

(2) In the robustness test, this paper measures Dig based on digitization-related intangible assets as a replacement variable [37]. First, this paper collects detailed data on the intangible assets of Chinese A-share-listed manufacturing companies. Second, based on De thesaurus constructed after Python text analysis in the previous section, the digitization-related part of the intangible assets of Chinese listed manufacturing companies is screened by matching. Finally, the ratio of the amount of digitization-related intangible assets to the total intangible assets of enterprises is calculated and used as a variable to measure Dig.

### Intermediate variables

1. Technological innovation (Tec) is expressed as the intensity of enterprise R&D investment, specifically measured by the ratio of R&D investment to operating income [38]. Dig helps enterprises improve their internal and external production factor management capabilities, reduce the expenditure on input factors such as manpower and raw materials, and thus increase the proportion of R&D investment [29]. The increase in R&D investment leads to an increase in the level of technological innovation of the enterprise, which helps the enterprise to improve the efficiency of resource allocation and utilization, improve the structure of energy consumption, and ultimately improve Gtf [53]. As a result, technological innovation can reflect the level of enterprise technological innovation, and we introduce technological innovation to study the channel mechanism of Dig through technological innovation to promote Gtf proposed in Hypothesis 2.
2. Financing constraint (Fin) is expressed as the ratio of interest expense to total debt [54]. Information asymmetry is an important cause of financial problems in manufacturing enterprises. Enterprises tend to reduce or even not disclose relevant information in order to protect their green innovations and enhance market competitiveness, which further enhances the information asymmetry problem [55]. While Dig helps to strengthen the docking efficiency between enterprises and investors and transmit enterprise-related information outward at low cost, which effectively alleviates the information asymmetry problem with investors and improves the speed and efficiency of financing [37], Therefore, we introduce financing constraint to explore the channel mechanism of Dig to promote Gtf by mitigating Fin as proposed in Hypothesis 3.

### Control variables

(1) Environmental regulation (Er) is selected as the ratio of the total investment in pollution control of each province in China to the total investment in pollution control of the whole country in that year [35]. Environmental regulation is an important regulatory approach for the government to urge Gtf. On the one hand, environmental regulation can prompt enterprises to optimize the structure of energy use and improve the efficiency of energy use. On the other hand, however, overly stringent regulation may lead to a decline in firms’ economic efficiency, thus affecting overall efficiency. However, to summarize, environmental regulation will have a corresponding impact on Gtf, and this paper takes environmental regulation as a control variable.

In the robustness test, this paper changes the measurement of Er by using the Python text analysis method [54]. First, this paper constructs an authoritative and comprehensive Er word collection. Eleven government work reports of the State Council of China from 2010 to 2020 were extracted as the source database of Er words. The 11 government work reports were put into Python for text segmentation, and 16 environment-related words that did not exist in the original Python thesaurus were added to the "jieba" thesaurus through manual identification. Then, the new thesaurus was used to perform word division and word frequency statistics on the 11 government work reports, and 56 Er words with a word frequency greater than 5 times were selected to form the Er word collection. Secondly, the textual analysis of the government work reports of each prefecture-level city was carried out. The government work reports of 281 prefectural cities from 2010 to 2020 were extracted, and the textual word frequency analysis of the government work reports of each prefectural city in each year was done using the selected 56 new Er thesaurus, in which the frequency of occurrence of the 56 Er words was counted. Finally, considering the differences in the length of each government work report, this paper takes the sum of the frequency of Er words divided by the total length of the government work report to express the Er value of the city in that year. Considering again the convenience of data expression, this paper multiplies the obtained ratio by 100 to express the final Er index.

(2) The level of urban development (Urb) is measured by GDP per capita [54], which can reflect the requirements of the government and the people for environmental quality. Based on the differences in urban geographic location and resource endowment, it may have a differentiated impact on Gtf in different regional contexts. On the one hand, people in higher Urb regions have higher demands for environmental quality and can afford the green innovation premium, which can effectively promote Gtf. On the other hand, higher Urb regions have greater infrastructure and resource advantages that can enhance the efficiency of green transformation. Therefore, Urb is expected to have a positive impact on Gtf.

(3) Enterprise age (Age) is measured by enterprise establishment time [54]. The year of enterprise establishment reflects the degree of dependence on traditional modes of production and operation and is closely related to innovation consciousness, which also affects Gtf [56]. Manufacturing enterprises established earlier have higher dependence on the traditional high-consumption and high-pollution production modes, higher green transition costs, and a weaker willingness to innovate. While enterprises established later in time have lower dependence on the path of traditional modes, higher flexibility, and are more helpful to Gtf. Therefore, it is necessary to control for the effect of age on Gtf when testing the effect of Dig on Gtf.

(4) Firm size (Siz) is expressed as the logarithm of total assets at the end of the year [37]. On the one hand, the larger Siz is, the more likely it is to provide a continuous and stable cash flow for the development of production technology innovation to support Gtf [56]. On the other hand, enterprises with too large Siz have basically fixed production patterns; the cost of production materials and technology substitution is too high; the strategic adjustment is more difficult; and more obstacles are encountered in the process of Gtf. At present, there is no unified conclusion on the impact of Siz on Gtf.

(5) Corporate financial leverage (Lev) is measured by the ratio of total liabilities to total assets [37]. Lev is an important indicator of a company’s future development potential, reflecting the company’s financial health. Based on the principles of profitability and safety, investors are more willing to invest their funds in enterprises with a lower LEV to reduce investment risks. The better the financial condition of the enterprise, the more it helps the enterprise to transform and upgrade, carry out green technological innovation, and promote green transformation. Therefore, it is necessary to control the effect of Lev on Gtf when testing the effect of Dig on Gtf.

(6) The corporate rate of return (Roa) is measured by the ratio of net profit to total assets at the end of the year [37]. Roa is a measure of the profitability of the enterprise, which reflects the amount of funds that can be used by the enterprise for green technology innovation. The stronger the profitability, the more adequate the enterprise’s own funds are, and the more capable it is to invest the funds in green technology innovation activities [57]. Therefore, Roa has a facilitating effect on Gtf, which is considered a control variable in this paper.

The main variables are described in Table 1:

**Table 1:** Description of main variables.

Variables	Variable name	Symbol	Variable specification
Explained variables	The level of green transformation of manufacturing enterprises	Gtf	Business income per unit of carbon emissions
Explanatory variables	Enterprise digitalization level	Dig	The total word frequency of corporate digital-related terms divided by the length of the MD&A segment of the annual report and multiplied by 100
Mediating variables	Technological Innovation	Tec	R&D investment/operating income
	Financing constraints	Fin	Interest expense/total liabilities
Control variables	Environmental Regulation	Er	The total investment in pollution control in each province in the current year is measured as a proportion of the total investment in pollution control in the country
	Level of urban development	Urb	Urban GDP per capita
	Number of years in business	Age	Enterprise establishment time
	Enterprise size	Siz	Logarithm of total assets at the end of the year
	Financial leverage	Lev	Total liabilities / total assets
	Yield	Roa	Net income / Total assets at year-end

### Data and descriptive statistics

This paper takes Chinese A-share listed companies in the manufacturing industry from 2010 to 2020 as the initial research sample and screens the samples according to the following principles: (1) excluding samples of ST and *ST companies; (2) interpolating the samples with missing relevant variables. Macronational, provincial, and city-level related data are mainly from the China Statistical Yearbook, the China City Statistical Yearbook, and the China Energy Statistical Yearbook, while microenterprise-level data are mainly from the Cathay Pacific database, the Wind database, and the Carbon Emission Trading Network.

Table 2 reports the descriptive statistics of the main variables. The mean value of Gtf is 4.652, implying that listed Chinese manufacturing companies generate an average of 465 million yuan of operating income per 10,000 tons of carbon emissions. The mean value of Dig is 0.787, suggesting that an average of 0.787% of the terms in the MD&A section of the annual reports of listed Chinese manufacturing companies are related to digitization. The maximum value is 4.443, which means that the maximum 4.443% of words in the MD&A section of annual reports are related to digitization. It can be seen that among the listed companies in China’s manufacturing industry, there are large differences in digitization among different companies. The distribution characteristics of the remaining variables are basically similar to those of previous studies and will not be repeated.

**Table 2:** Descriptive statistics of main variables.

Variables	Symbol	Number of samples	Mean	Std.Dev	Min	Max
Dependent variable	Gtf	8646	4.652	6.265	-318.815	46.221
Core explanatory variables	Dig	8387	0.787	0.527	0.037	4.443
Mediating variables	Tec	8335	4.246	4.367	0.000	137.450
	Fin	8025	0.020	0.036	-0.499	2.767
Control variables	Siz	8652	3.717	1.204	-2.161	9.126
	Lev	8679	0.403	0.207	-0.518	2.849
	Roa	8679	0.041	0.088	-3.200	1.408
	Urb	7309	2.253	0.479	0.055	3.845
	Er	8349	0.052	0.037	0.000	0.166
	Age	8679	17.332	5.891	0.000	65.000

## Results

### Benchmark regression, endogeneity test and robustness test

#### Benchmark regression results

Table 3 presents the baseline regression of the impact of Dig on Gtf. Column (1) directly regresses the two and finds that the coefficient of Dig is 4.681, which is significantly positive at the 1% level, but the R2 is only 0.229, which is not a good model fit. Columns (2) to (7) gradually added the corresponding control variables, and the data show that the coefficient of Dig is still significantly positive at the 1% level. The R2 increased from 0.229 to 0.272; although there is an increase, the fitting effect is still at a poor level. Columns (8) through (10) progressively control for time, province, and industry fixed effects with the addition of control variables. As shown in Column (10), the coefficient of Dig is 0.675 at this time, which is significantly positive at the 1% level, and the R2 is improved to 0.727. The above results gradually verify the facilitating effect of Dig on Gtf, and Hypothesis 1 is verified.

**Table 3:** Benchmark regression results.

	(1)	(2)	(3)	(4)	(5)	(6)	(7)	(8)	(9)	(10)
	Gtf	Gtf	Gtf	Gtf	Gtf	Gtf	Gtf	Gtf	Gtf	Gtf
Dig	4.681*** (39.857)	4.679*** (39.822)	4.647*** (39.662)	4.634*** (39.606)	4.191*** (33.665)	4.127*** (32.853)	4.127*** (33.256)	3.680*** (31.243)	3.508*** (29.359)	0.675*** (7.056)
Siz		***	0.309*** (6.762)	0.336*** (7.164)	0.221*** (4.316)	0.278*** (5.261)	0.187*** (3.485)	-0.129** (-2.399)	-0.112** (-2.051)	-0.144*** (-3.820)
Lev			-2.948*** (-10.675)	-3.252*** (-10.652)	-2.878*** (-8.579)	-2.992*** (-8.695)	-3.186*** (-9.358)	-2.260*** (-6.906)	-2.169*** (-6.720)	-1.287*** (-5.674)
Roa				-1.449** (-2.292)	-1.892*** (-2.577)	-1.941*** (-2.643)	-1.518** (-2.025)	0.650 (0.805)	0.817 (1.019)	2.481*** (3.056)
Urb					1.831*** (15.981)	1.932*** (16.337)	1.761*** (14.796)	1.194*** (10.343)	0.863*** (6.537)	0.080 (0.884)
Er						-9.011*** (-6.201)	-9.598*** (-6.660)	-12.776*** (-8.907)	-5.835* (-1.711)	-3.468* (-1.691)
Age							0.100*** (9.331)	-0.017 (-1.494)	-0.027** (-2.258)	-0.030*** (-3.947)
Year FE	NO	NO	NO	NO	NO	NO	NO	YES	YES	YES
Porv FE	NO	NO	NO	NO	NO	NO	NO	NO	YES	YES
Industy FE	NO	NO	NO	NO	NO	NO	NO	NO	NO	YES
N	8355	8354	8354	8354	7024	6778	6778	6778	6778	6778
R^2^	0.229	0.229	0.240	0.240	0.258	0.261	0.272	0.336	0.357	0.727

Note:*** p<0.01

** p<0.05

* p<0.1.t-statistics in parentheses. Unless otherwise noted, the parentheses in the table below are the same as here.

#### Endogeneity test

Potential endogeneity issues could cast doubt on the results of the previous study. Dig may facilitate Gtf. Meanwhile, firms with a higher degree of greening may themselves have a stronger willingness to adopt digital technologies and actively promote Dig, leading to simultaneity bias issues. In addition, issues such as modeling bias or the omission of variables may also lead to endogeneity. For example, factors that simultaneously affect Dig and Gtf may be omitted. To mitigate the endogeneity problem, on the one hand, we control the time, industry, and province-level characteristic factors in the baseline model. On the other hand, this paper employs the lagged variable approach, instrumental variable approach, and exogenous shock event test to mitigate the adverse effects of the endogeneity problem on the research findings even further.

(1) Quoting lagged terms for explanatory variables. On the one hand, the problem of simultaneity bias can be mitigated to some extent by applying lagged one-period treatment to all explanatory variables of the benchmark model. The results are shown in columns (1) to (4) of Table 4. On the other hand, the core explanatory variables are lagged by one or two periods and then regressed again. The results are shown in Table 4, columns (5), and (6). The results reported in Table 4 show that the impact of Dig on their carbon efficiency shows a significant positive effect, whether lagged by one or two periods.

**Table 4:** Endogeneity test—variable lag method.

	(1)	(2)	(3)	(4)	(5)	(6)
	Gtf	Gtf	Gtf	Gtf	Gtf	Gtf
L.Dig	4.375*** (32.139)	3.925*** (29.965)	3.747*** (28.084)	0.680*** (6.461)	0.713*** (6.778)	
L2.Dig						0.681*** (5.918)
Controls	YES	YES	YES	YES	YES	YES
Year FE	NO	YES	YES	YES	YES	YES
Industy FE	NO	NO	YES	YES	YES	YES
Porv FE	NO	NO	NO	YES	YES	YES
N	6152	6152	6152	6152	6136	5488
R^2^	0.274	0.326	0.348	0.741	0.741	0.761

(2) Invoking instrumental variables. Instrumental variables are an important means to solve the endogeneity problem. Generally speaking, instrumental variables need to fulfill two conditions: one is the correlation with endogenous explanatory variables, and the other is exogenous. A large number of scholars select the lagged period of endogenous variables as their instrumental variables [58, 59].

Referring to previous studies, this paper chooses the first-order lag term of Dig as the instrumental variable. On the one hand, Dig is inseparable from its digitization level in the previous period, which meets the requirement of instrumental variable relevance. On the other hand, since the Dig of the previous period is a historical variable, it has little correlation with other factors affecting the carbon efficiency level of enterprises in the current period.

In this paper, we use two-stage least squares (2SLS) for estimation. Meanwhile, in order to test the robustness of the results, we refer to James as well as He et al. and add control variables one by one [60, 61]. The results are shown in Table 5, columns (1) to (7).

**Table 5:** Endogeneity test—instrumental variable approach.

	(1)	(2)	(3)	(4)	(5)	(6)	(7)
	Gtf	Gtf	Gtf	Gtf	Gtf	Gtf	Gtf
Dig	0.812*** (7.171)	0.844*** (7.519)	0.856*** (7.686)	0.861*** (7.757)	0.850*** (7.222)	0.833*** (7.011)	0.811*** (6.802)
Siz		-0.173*** (-5.443)	-0.027 (-0.845)	-0.081** (-2.363)	-0.148*** (-3.919)	-0.169*** (-4.292)	-0.163*** (-4.161)
Lev			-1.899*** (-10.751)	-1.400*** (-6.971)	-1.458*** (-6.222)	-1.467*** (-6.144)	-1.370*** (-5.702)
Roa				2.268*** (3.710)	2.395*** (2.856)	2.408*** (2.842)	2.429*** (2.908)
Urb					0.077 (0.815)	0.041 (0.422)	0.018 (0.188)
Er						-0.059 (-0.027)	-0.061 (-0.027)
Age							-0.030*** (-3.867)
Year FE	YES	YES	YES	YES	YES	YES	YES
Porv FE	YES	YES	YES	YES	YES	YES	YES
Industy FE	YES	YES	YES	YES	YES	YES	YES
First stAge F-test	8546.29***	8500.81***	8474.75***	8501.72***	7358.68***	7164.14***	7123.62***
Kleibergen-Paap rk LM statistics	1116.405***	1129.008***	1129.406***	1129.606***	1016.613***	995.677***	995.693***
N	7569	7569	7569	7569	6360	6136	6136
R^2^	0.738	0.739	0.743	0.744	0.741	0.740	0.741

Note:*** p<0.01

** p<0.05

* p<0.1.z-statistics in parentheses.

As can be seen in Table 5, on the one hand, the Kleibergen-Paap rk LM statistics in all seven equations significantly reject the null hypothesis of under-identification, proving the correlation between instrumental and endogenous variables. On the other hand, the F-statistics of the first-stage regressions in all seven equations are greater than 10, rejecting the null hypothesis of weak instrumental variables. The above results fully prove the validity of using instrumental variables in this paper [62].

Under the premise of the validity of instrumental variables, Table 5 fully demonstrates the significant role of Dig in influencing its carbon efficiency. As shown in column (7) of Table 5, the coefficient of Dig is 0.811, indicating that for every 1% increase in the digitization degree of a firm, the carbon efficiency level of the firm will increase by 0.811%.

(3) Invoking exogenous shock events. The broadband China policy is introduced as a proxy variable for Dig, and the regression results are shown in Table 6. The validity of the DID results depends on the parallel trend hypothesis test. According to model (3), we use the event study method to conduct the parallel trend test, and the event study model can provide the dynamic impact of broadband China policy on corporate carbon efficiency [44].

**Table 6:** Endogeneity test—exogenous shock event test method.

	(1)	(2)	(3)	(4)
	Gtf	Gtf	Gtf	Gtf
DID	3.495*** (23.788)	1.069*** (4.947)	1.179*** (5.090)	0.507*** (2.657)
Controls	NO	YES	YES	YES
Year FE	NO	YES	YES	YES
Porv FE	NO	NO	YES	YES
Industy FE	NO	NO	NO	YES
N	7491	6973	6973	6973
R^2^	0.070	0.144	0.166	0.453

**Fig 2:**
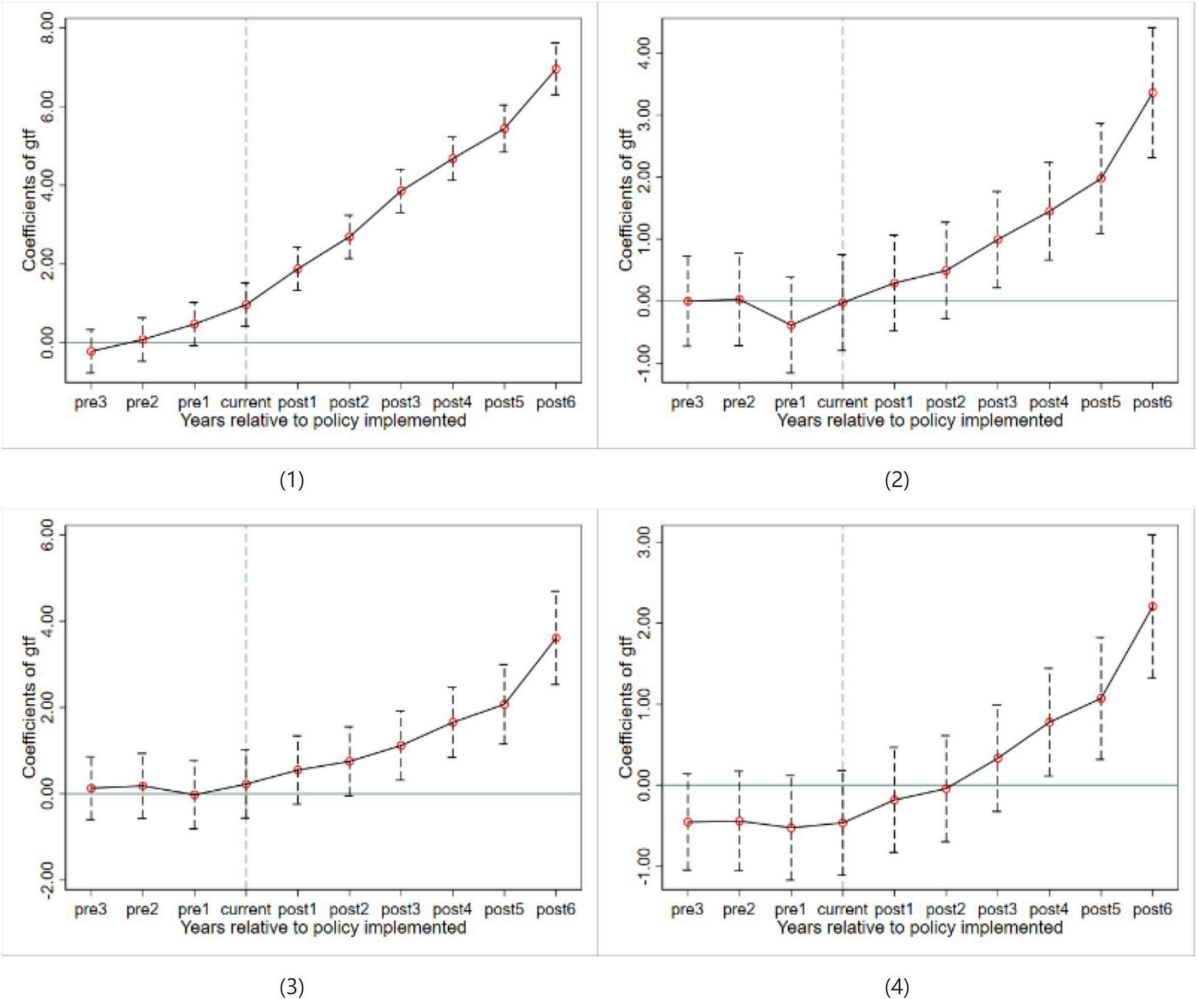
DID parallel trend test and dynamic test for columns (1) through (4) of Table 6.

Fig 2 shows the parallel trend hypothesis testing for the four DID regression equations in Table 6. The results show that there was no statistical difference between the carbon efficiency level of enterprises in pilot cities and non-pilot cities, and the model met the parallel trend hypothesis.

Combined with Table 6 columns and Fig 2, it can be seen that, from the average impact effect, the policy shock has a significant promotion effect on Gtf. From the dynamic impact effect, there is a time lag in the impact of policy on Gtf. In terms of column (4) of Table 6, the policy effect gradually shows its impact after 3 years of implementation and is not significant in the year of implementation and the following year.

### Other robustness tests

(1) Change the measure of the dependent variable. The capital input is measured by the total fixed assets of the enterprise; the labor input is measured by the number of employees of the enterprise; the business revenue is defined as the desired output; and the carbon emission of the enterprise is defined as the non-desired output. The super-efficiency SBM-GML model as well as the EBM-GML model are used to measure Gtf. The results are shown in Table 7.

**Table 7:** Robustness tests—replacement of dependent variables.

	(1)	(2)	(3)	(4)	(5)	(6)	(7)	(8)	(9)	(10)
	Gtf3	Gtf3	Gtf3	Gtf3	Gtf3	Gtf4	Gtf4	Gtf4	Gtf4	Gtf4
Dig	0.037*** (14.470)	0.033*** (12.502)	0.030*** (11.712)	0.030*** (12.003)	0.018*** (6.958)	0.051*** (14.662)	0.046*** (12.997)	0.044*** (12.884)	0.045*** (13.043)	0.022*** (6.168)
Controls	NO	YES	YES	YES	YES	NO	YES	YES	YES	YES
Year FE	NO	NO	YES	YES	YES	NO	NO	YES	YES	YES
Porv FE	NO	NO	NO	YES	YES	NO	NO	NO	YES	YES
Industy FE	NO	NO	NO	NO	YES	NO	NO	NO	NO	YES
N	6533	5291	5291	5291	5291	6531	5289	5289	5289	5289
R^2^	0.061	0.148	0.177	0.194	0.249	0.063	0.131	0.152	0.167	0.237

Columns (1) to (5) of Table 7 show the regression results after replacing the original dependent variables with the corporate carbon efficiency constructed by the EBM-GML model. Dig consistently showed a significant positive effect. Columns (6) to (10) of Table 7 show the regression results after replacing the original dependent variables with corporate carbon efficiency constructed by the SBM-GML model. After the replacement of variables, control variables and fixed effects are added gradually. Dig also consistently showed a significant positive effect.

(2) Change the measurement of Dig and control variables. On the one hand, Dig is measured based on digitization-related intangible assets, and control variables and fixed effects are gradually added for robustness testing. The results are shown in columns (1) to (5) of Table 8. On the other hand, the measurement of environmental regulation variables in the control variables is changed. The results are shown in column (6) of Table 8.

**Table 8:** Robustness tests—changing the core explanatory variables and control variables.

	(1)	(2)	(3)	(4)	(5)	(6)
	Gtf	Gtf	Gtf	Gtf	Gtf	Gtf
Dig2	17.661*** (12.141)	14.202*** (9.337)	12.591*** (8.518)	11.281*** (8.011)	2.571* (1.721)	
Dig						0.680*** (7.133)
Controls	NO	YES	YES	YES	YES	YES
Year FE	NO	NO	YES	YES	YES	YES
Industy FE	NO	NO	NO	YES	YES	YES
Porv FE	NO	NO	NO	NO	YES	YES
N	8646	7005	7005	7005	7005	6912
R^2^	0.044	0.103	0.162	0.181	0.453	0.728

The coefficients of Dig are always positive and significant after the change in measurement of Dig and the control variables. The data support the conclusion that Dig has a significant facilitating effect on Gtf.

Through a series of endogeneity and robustness tests such as lagged variables, the introduction of instrumental variables, the introduction of exogenous policy shocks, the replacement of indicators, etc., the above adequately demonstrates the significant facilitating effect of Dig on the improvement of its carbon efficiency level from various perspectives. It confirms that Dig has a significant positive facilitating effect on Gtf. The reliability and robustness of Hypothesis 1 are fully demonstrated.

### Channels and mechanisms analysis

This section empirically tests the impact mechanism using a mediated effects model.

The estimation results in column (2) of Table 9 indicate that technological innovation increases significantly with the level of Dig. Column (3) of Table 9 reports the mediation effect test for technological innovation. It can be seen that the estimated coefficient of R&D investment intensity is significantly positive, while Dig remains significant. It indicates that Dig promotes Gtf by improving technological innovation. Hypothesis 2 is verified.

**Table 9:** Mechanistic test of digital transformation on carbon efficiency.

	(1)	(2)	(3)	(4)	(5)	(6)
	Gtf	Tec	Gtf	Gtf	Fin	Gtf
Dig	0.675*** (7.056)	1.917*** (12.657)	0.439*** (3.846)	0.675*** (7.056)	-0.001*** (-3.464)	0.748*** (7.802)
Tec			0.120*** (3.859)			
Fin						-4.280* (-1.933)
Controls	YES	YES	YES	YES	YES	YES
Year FE	YES	YES	YES	YES	YES	YES
Industy FE	YES	YES	YES	YES	YES	YES
Porv FE	YES	YES	YES	YES	YES	YES
N	6778	6764	6733	6778	6497	6466
R^2^	0.727	0.277	0.735	0.727	0.181	0.741

The estimation results in column (5) of Table 9 indicate that the financing constraints of enterprises are alleviated with the improvement of Dig. Column (6) of Table 9 reports the mediation effect test of financing constraints. The estimated coefficient of financing constraints is significantly negative, while the digitization variable remains significant. It indicates that Dig promotes Gtf by alleviating financing constraints. Hypothesis 3 is verified.

### Nonlinear spillover effects

Table 10 presents the results of the test for threshold effects, and Table 11 reports the threshold model. Combining the analysis of Tables 10 and 11, several conclusions can be drawn. First, the full sample and eastern China passed the double threshold test. Dig has a significant dynamic nonlinear spillover effect on the process of promoting Gtf and shows a significantly decreasing marginal effect. Hypothesis 4 is verified. Second, on the one hand, central China passed the double-threshold test, but its regression results are not significant, and the sign of the regression coefficient of Dig on Gtf is inconsistent with the economic meaning. On the other hand, western and northeastern China did not pass the threshold test. This indicates that the nonlinear spillover effect is not effectively utilized in the central, western, and northeastern regions of China.

**Table 10:** Tests for digitization threshold effects.

Region	Threshold variable	Model	F-statistic	p-value	Threshold value		
					10%	5%	1%
All Cities	Dig	Single Threshold	79.81	0.000	9.091	10.389	16.307
		Double Threshold	42.43	0.000	10.231	11.915	15.091
		Triple Threshold	17.80	0.513	37.486	45.404	58.229
East	Dig	Single Threshold	70.15	0.000	9.769	12.285	16.714
		Double Threshold	24.04	0.000	10.013	11.480	15.507
		Triple Threshold	14.32	0.343	20.403	22.224	27.305
West	Dig	Single Threshold	10.04	0.303	17.745	22.867	27.171
		Double Threshold	4.45	0.587	17.716	22.170	30.657
		Triple Threshold	4.89	0.573	16.521	20.784	32.000
Central	Dig	Single Threshold	31.71	0.000	9.844	11.608	19.380
		Double Threshold	16.19	0.100	15.454	24.978	46.002
		Triple Threshold	10.30	0.350	20.213	31.819	47.878
Northeast	Dig	Single Threshold	6.02	0.307	8.799	10.463	15.916
		Double Threshold	7.56	0.210	9.693	10.910	13.815
		Triple Threshold	6.02	0.380	10.851	12.450	16.446

**Table 11:** Digitization threshold model regression results.

	All Cities			East			Central		
	Gtf(1)	Gtf(2)	Gtf(3)	Gtf(4)	Gtf(5)	Gtf(6)	Gtf(7)	Gtf(8)	Gtf(9)
Threshold value	Dig≤	0.346<Dig≤1.658	Dig>1.658	Dig≤	0.360<Dig≤1.583	Dig>1.583	Dig≤	0.788<Dig≤1.914	Dig>1.914
Dig	3.292*** (6.01)	0.743*** (2.67)	1.482*** (5.94)	2.977*** (4.71)	0.661** (2.05)	1.480*** (5.44)	-2.596** (-2.53)	-1.132* (-1.68)	0.303 (0.43)

### Heterogeneity analysis

#### Heterogeneity test based on micro characteristics of firms

First, the sample is divided into state-owned enterprises and non-state-owned enterprises based on the nature of enterprise ownership. Second, with reference to the Measures for the Division of Statistically Large, Small, Medium, and Micro Enterprises (2017) issued by the China Statistical Bureau, the sample is divided into large enterprises and small and medium-sized enterprises based on the number of employees and business revenues of the enterprises in the year. The regression results are shown in Table 12.

**Table 12:** Heterogeneity test based on micro characteristics of firms.

	(1)	(2)	(3)	(4)
	Gtf	Gtf	Gtf	Gtf
Dig	0.818*** (5.309)	0.662*** (5.329)	0.714*** (7.039)	0.602** (2.058)
Controls	YES	YES	YES	YES
Year FE	YES	YES	YES	YES
Industy FE	YES	YES	YES	YES
Porv FE	YES	YES	YES	YES
N	2550	4228	5802	976
R^2^	0.774	0.720	0.763	0.658

From the results in columns (1) and (2) of Table 12, it can be seen that Dig significantly promotes Gtf, both for state-owned and non-state-owned enterprises. Among them, the promotion effect of state-owned enterprises is more obvious. The possible reason for this result is that Dig requires large investments in digital technology, smart manufacturing, and information systems, and Chinese SOEs have more advantages in terms of capital, scale, research, and policies. From the data, it seems that Chinese SOEs are fully seizing the windfall of Dig to make up for their efficiency loss and move towards high-quality development.

The results in columns (3) and (4) of Table 12 show that Dig significantly contributes to Gtf. Among them, the promotion effect of large enterprises is more obvious. This result suggests that Dig has a scale effect, and the larger the enterprise, the easier it is to exert the effect of digitalization.

### Heterogeneity test based on macro characteristics of cities

(1) Direct effect heterogeneity test. At the macro level, the heterogeneity is analyzed in terms of two characteristics of Chinese urban agglomerations and city levels. On the one hand, Beijing-Tianjin-Hebei Delta, Yangtze River Delta, Pearl River Delta, Middle Yangtze River, Central Plains Urban Agglomeration, and Chengdu-Chongqing Urban Agglomeration are categorized as a sample of key urban agglomerations, and the others are non-focused. The test results are shown in columns (1) and (2) of Table 13. On the other hand, China’s Tier 1, New Tier 1, Tier 2, and Tier 3 cities are categorized as high-level cities, and the rest are low-level cities. The test results are shown in column (3) and column (4) of Table 13.

**Table 13:** Heterogeneity test based on macro characteristics of cities.

	(1)	(2)	(3)	(4)
	Gtf	Gtf	Gtf	Gtf
Dig	0.661*** (6.622)	0.114 (0.344)	0.726*** (6.698)	0.559** (2.519)
Controls	YES	YES	YES	YES
Year FE	YES	YES	YES	YES
Industy FE	YES	YES	YES	YES
Porv FE	YES	YES	YES	YES
N	6057	721	5043	1735
R^2^	0.724	0.796	0.720	0.770

The results show that, from a city cluster perspective, the impact of Dig on Gtf is significantly positive in the key city clusters, but the effect is not significant in other cities. From a city-level perspective, the effect of Dig on Gtf is significantly positive in both high-level and low-level cities, but the release of dividends from Dig is more fully realized among enterprises in high-level cities.

A plausible explanation for this phenomenon is that key city clusters and high-level cities have relatively developed infrastructures and a wide range of cutting-edge digital technologies, and local firms can utilize these first-mover advantages to empower their own systems. On the contrary, there is a considerable gap between the digital endowment in non-focused city clusters and low-level cities and developed regions. The green effect of Dig on enterprises in this part of the region is not obvious. This shows that the problem of uneven development between different regions is also reflected at the local enterprise level.

(2) Heterogeneity test for nonlinear spillover effects. Combined with the analysis of Tables 14 and 15, the following conclusions can be obtained: First, key city clusters and high-level cities have passed the double threshold test. Dig has a significant dynamic nonlinear spillover effect on the process of promoting Gtf, and the effect shows a significantly decreasing marginal effect. Second, on the one hand, non-focused city clusters passed the single-threshold test, but their regression results were not significant. On the other hand, low-level cities did not pass the threshold test. It indicates that the nonlinear spillover effect is not effectively utilized in the enterprises of non-focused cities and low-level cities.

**Table 14:** Heterogeneity analysis of threshold effect tests.

Region	Threshold variable	Model	F-statistic	p-value	Threshold value		
					10%	5%	1%
Key city clusters	Dig	Single Threshold	63.96	0.000	10.245	11.129	14.562
		Double Threshold	37.74	0.000	8.841	10.588	15.245
		Triple Threshold	10.41	0.583	21.851	24.349	30.402
Non-focused city groups	Dig	Single Threshold	16.88	0.003	9.216	10.390	14.176
		Double Threshold	10.08	0.163	11.983	16.440	23.532
		Triple Threshold	5.67	0.603	16.115	20.733	28.114
High-level cities	Dig	Single Threshold	70.42	0.000	9.254	11.137	13.203
		Double Threshold	35.52	0.010	10.701	12.904	17.991
		Triple Threshold	15.46	0.550	33.162	43.255	54.513
Low-level cities	Dig	Single Threshold	8.06	0.140	8.865	12.382	17.057
		Double Threshold	3.95	0.540	10.478	12.827	16.822
		Triple Threshold	2.25	0.760	9.983	13.589	19.397

**Table 15:** Heterogeneity threshold model regression results.

	Key city clusters			Non-key city clusters		High level cities		
	Gtf(1)	Gtf(2)	Gtf(3)	Gtf(4)	Gtf(5)	Gtf(6)	Gtf(7)	Gtf(8)
Threshold value	Dig≤	0.345<Dig≤1.593	Dig>1.593	Dig≤	Dig>1.433	Dig≤	0.346<Dig≤1.700	Dig>1.700
Dig	3.847*** (5.63)	0.817** (2.52)	1.560*** (5.64)	-0.392 (-0.67)	0.429 (0.94)	3.328*** (5.78)	0.788*** (2.81)	1.510*** (5.86)

## Discussion

Based on the fact that digital technology has affected Gtf, this paper takes China as an example, and from the micro perspective of manufacturing enterprises, based on the data of Chinese A-share listed companies from 2010 to 2020, based on the use of python text analysis method to portray the digitization level of Chinese manufacturing enterprises, it systematically examines the impact of Dig on Gtf.

The findings show that Dig can significantly promote Gtf, and this conclusion still holds after a series of endogeneity tests and robustness tests. The analysis of channels and mechanisms finds that Dig can promote Gtf through two channels: enhancing technological innovation and alleviating financing constraints. Further research also finds that the impact of Dig on Gtf has a positive nonlinear spillover effect, but its marginal effect is decreasing. Finally, the impact of Dig on Gtf has obvious heterogeneous effects: from the viewpoint of enterprise micro characteristics, Dig promotes the Gtf of state-owned enterprises (SOEs) and large-scale enterprises (LSEs) more. From the analysis of urban macro characteristics, whether from the direct effect or non-linear spillover effect, Gtf effect promoted by the release of Dig dividend is greater in the eastern region, key urban agglomerations and high-level cities.

Based on the above findings, this paper has the following policy implications for developed countries to "revitalize" the manufacturing industry and developing countries to promote the manufacturing industry from big to strong.

First, accelerate the development of Dig. Whether from the competition of the new round of scientific and technological revolution, or the competition of the global value chain division of labor status, accelerate the promotion of digital technology progress and development, constructing a first-mover advantage is the urgent need of the world’s countries.

Second, create an open-source digital technology progress pattern. Full participation in the international exchange of digital technology is an effective way for global manufacturing enterprises to realize green transformation faster. At present, the spread of the epidemic, "anti-globalization" point of view revival, the healthy development of the world economy is hindered, but the more difficult it is the more the test of the country and the enterprise strategy boldness time. Take China as an example, in December 2022, under the epidemic, the Zhejiang Provincial Department of Commerce and other relevant provincial government departments led Chinese enterprise representatives still went to Europe to open a six-day business trip, demonstrating the will and determination of China and Chinese enterprises to open up to the outside world.

Third, integrating financial resources and developing financing channels on the premise of risk prevention. For example, combining blockchain and other technological methods to create an online financing platform with higher efficiency and lower risk is a suitable route. Easing the financing constraints of manufacturing enterprises is an important way to unleash their technological innovation and promote the industry into high-quality green development.

Fourth, fully recognize the digital divide that exists between regions. There are differences in the level of Dig and the stage of Gtf in different regions. On the one hand, all regions and enterprises in each country should combine their comparative advantages according to their own actual situation to enhance their core characteristic competitiveness. On the other hand, they should make full use of the non-linear spillover effect of digital technology to strengthen win-win cooperation with other countries and regions for synergistic development.

Fifthly, it is necessary to continuously improve digital governance capacity and dynamically monitor the potential risks arising from digital technology.
